# Mathematical model of insulin kinetics accounting for the amino acids effect during a mixed meal tolerance test

**DOI:** 10.3389/fendo.2022.966305

**Published:** 2022-09-15

**Authors:** Micaela Morettini, Maria Concetta Palumbo, Christian Göbl, Laura Burattini, Yanislava Karusheva, Michael Roden, Giovanni Pacini, Andrea Tura

**Affiliations:** ^1^ Department of Information Engineering, Università Politecnica delle Marche, Ancona, Italy; ^2^ CNR Institute for Applied Computing, Rome, Italy; ^3^ Department of Obstetrics and Gynecology, Medical University of Vienna, Vienna, Austria; ^4^ Institute for Clinical Diabetology, German Diabetes Center, Leibniz Center for Diabetes Research, Düsseldorf, Germany; ^5^ German Center for Diabetes Research, Partner Düsseldorf, Neuherberg, Germany; ^6^ Department of Endocrinology and Diabetology, Medical Faculty and University Hospital, Heinrich-Heine University, Düsseldorf, Germany; ^7^ Independent Researcher, Padova, Italy; ^8^ CNR Institute of Neuroscience, Padova, Italy

**Keywords:** branched-chain amino acids, insulin secretion, type 2 diabetes, minimal model, parameter estimation, glucose homeostasis

## Abstract

Amino acids (AAs) are well known to be involved in the regulation of glucose metabolism and, in particular, of insulin secretion. However, the effects of different AAs on insulin release and kinetics have not been completely elucidated. The aim of this study was to propose a mathematical model that includes the effect of AAs on insulin kinetics during a mixed meal tolerance test. To this aim, five different models were proposed and compared. Validation was performed using average data, derived from the scientific literature, regarding subjects with normal glucose tolerance (CNT) and with type 2 diabetes (T2D). From the average data of the CNT and T2D people, data for two virtual populations (100 for each group) were generated for further model validation. Among the five proposed models, a simple model including one first-order differential equation showed the best results in terms of model performance (best compromise between model structure parsimony, estimated parameters plausibility, and data fit accuracy). With regard to the contribution of AAs to insulin appearance/disappearance (k_AA_ model parameter), model analysis of the average data from the literature yielded 0.0247 (confidence interval, CI: 0.0168 – 0.0325) and -0.0048 (CI: -0.0281 – 0.0185) μU·ml^-1^/(μmol·l^-1^·min), for CNT and T2D, respectively. This suggests a positive effect of AAs on insulin secretion in CNT, and negligible effect in T2D. In conclusion, a simple model, including single first-order differential equation, may help to describe the possible AAs effects on insulin kinetics during a physiological metabolic test, and provide parameters that can be assessed in the single individuals.

## 1 Introduction

Amino acids (AAs) are well known to be involved in the regulation of glucose metabolism and especially insulin secretion (1). Different AAs may exert distinct effects on postprandial glucose and insulin concentrations, which can be affected by their co-ingestion with glucose (2). Recently, much attention has been devoted to the branched-chain amino acids (BCAAs), comprising leucine, isoleucine, and valine. They are essential AAs that can be taken only with diet, meat and dairy products being the main sources (3). Whole-body disposal of BCAAs mainly results from their catabolism in the skeletal muscle and altered BCAAs catabolism has been associated with several disorders, such as obesity and type 2 diabetes (4). Moreover, fasting and postprandial plasma levels of BCAAs have been found associated with greater insulin secretion and reduced insulin clearance in healthy humans (5). Also, short-term dietary reduction of BCAAs reduced meal-induced insulin secretion (6). On the other hand, in recent-onset diabetes, BCAAs were associated negatively with meal-induced insulin secretion (7). Thus, understanding the effects of different AAs on insulin release and kinetics under different metabolic conditions may help to gain further insight on the development of several diseases, including type 2 diabetes, and to develop therapeutic strategies based on appropriate dietary regimen.

Mathematical models may help to analyze these effects. However, to the best of our knowledge, only one attempt has been done in this direction, considering the effects of AAs on the electrical activity leading to insulin granule exocytosis, based on *in-vitro* experiments (8). A model describing the AAs effect on the insulin kinetics at whole body level during a physiological metabolic test, such as a mixed-meal tolerance test (MMTT), is still lacking. Thus, our aim is to propose a new mathematical model, describing insulin kinetics in plasma during a MMTT, which includes the effect of AAs. To this end, a series of mathematical models was developed, analyzed and compared, on the basis of previously published data (7). The resulting chosen model may help in characterizing the different role of AAs (or specific categories of them, such as BCAAs) on insulin secretion.

## 2 Materials and methods

### 2.1 Model formulation

**Figure 1:**
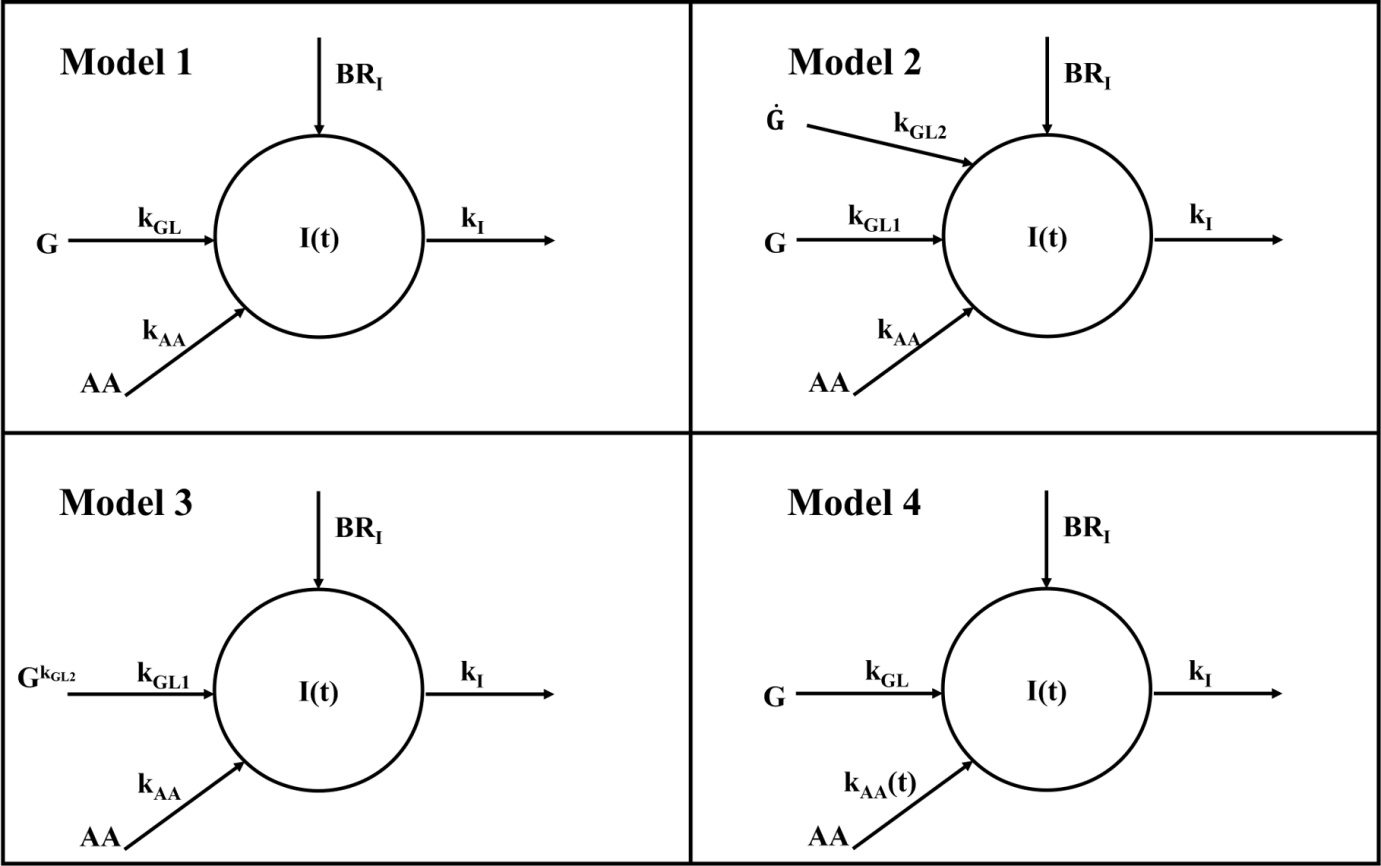
Compartmental representation of the four single-compartment model. All models are composed by a single compartment representing plasma insulin I(t). Plasma glucose G acts as input for all models through the k_GL_ parameter for Model 1 and 4 and k_GL1_ for Model 2 and 3; similarly, amino acids act as input for all models through k_AA_ parameter for Model 1, 2 and 3 and through the time-varying parameter k_AA_(t) for Model 4. In all models, k_I_ represents the insulin elimination rate from plasma and BR_I_ is the basal insulin appearance rate due to factors other than glucose and amino acids. In Model 2, a further dependence on glucose rise is included through the k_GL2_ parameter; in Model 2 this rise is accounted for by glucose derivative G˙ hereas in Model 3 by glucose exponentiation G(t)k^GL2^ .

**Figure 2:**
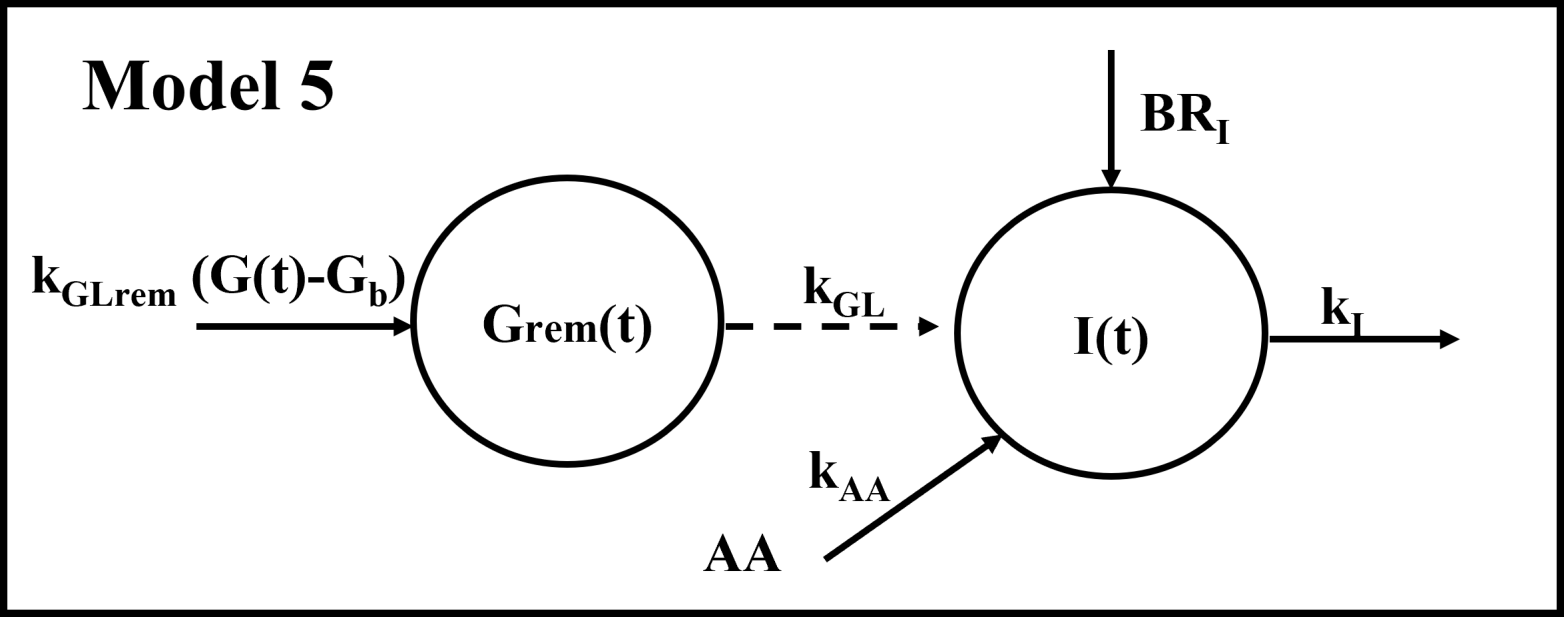
Compartmental representation of the double-compartment model. The model is composed by a compartment representing plasma insulin I(t) and a compartment representing glucose (suprabasal concentration) remote from plasma G_rem_(t). The input to the remote compartment is represented by suprabasal plasma glucose (G(t)-G_b_), through the k_GLrem_ parameter. G_rem_(t) acts as input through the k_GL_ parameter; similarly, amino acids act as input through the k_AA_ parameter; k_I_ represents the insulin elimination rate from plasma and BR_I_ is the basal insulin appearance rate due to factors other than glucose and amino acids.

We developed five different mathematical models. All models were based on the hypothesis that plasma insulin concentration during the metabolic test (MMTT) depends on the following contributions: (i) insulin appearance due to pancreatic secretion triggered by plasma glucose (main contribution); (ii) insulin appearance (or disappearance) due to the effect of AAs on pancreatic secretion (smaller contribution); (iii) basal insulin appearance rate due to factors other than MMTT glucose and AAs; (iv) insulin disappearance, mainly due to insulin utilization, insulin clearance from plasma, hepatic insulin extraction. Four out of five models are based on a single-compartment description (Model 1, 2, 3 and 4), whereas the remaining one (Model 5) comprises two compartments. In the single-compartment models, the compartment represents the plasma body space. In the two-compartment model, one compartment is again plasma, whereas the other one represents a different body space (not precisely identified from an anatomical point of view), which is remote from plasma. In all proposed models, plasma glucose and plasma AAs concentrations were used as inputs to the model; moreover, insulin disappearance was fixed to a constant value [0.14 min^-1^ (9)]. Compartmental structures for the four single-compartment models and the two-compartment model are reported in **Figures 1**, **2**, respectively.

#### 2.1.1 Model 1

Model 1 assumes that changes in plasma insulin concentration during a MMTT (I(t), μU·ml^-1^) are determined by plasma glucose (G(t), mg/dl) through a linear flux with transfer coefficient equal to k_GL_ (μU·ml^-1^/(mg·dl^-1^·min)), and by a second contribution, expected to be smaller than the first, due to the plasma AAs (AA(t), μmol·l^-1^), again supposed to be linear, with transfer coefficient equal to k_AA_ (μU·ml^-1^/(μmol·l^-1^·min)). Insulin clearance is assumed linear, with fractional transfer coefficient named k_I_ (min^-1^) equal to the fixed value indicated in the above paragraph. The constant contribution BR_I_ represents the basal insulin appearance rate due to factors other than glucose and AAs, and can be computed from steady-state conditions. Model 1 is described by the following differential equation:


$$\frac{dI(t)}{dt}=k_{GL}\cdot G(t)+k_{AA}\cdot AA(t)-k_{I}\cdot I(t)+BR_{I}$$

(1) $$I\text{(0)}=I_{b}$$

Plasma glucose and plasma AAs concentrations were used as inputs to the model; by imposing steady-state condition BR_I_ was assessed as follows:


(2) $$BR_{I}=-k_{GL}\cdot G_{b}-k_{AA}\cdot AA_{b}+k_{I}\cdot I_{b}$$

In the above equations, as well as in the following ones, the “*b*” subscript indicates the basal (fasting) value of one variable or parameter.

#### 2.1.2 Model 2

Model 2 assumes that changes in plasma insulin concentrations during a MMTT are determined by both plasma glucose G(t) and its rate of change dG(t)/dt, through k_GL1_ (μU·ml^-1^/(mg·dl^-1^·min)) and k_GL2_ (μU·ml^-1^/(mg·dl^-1^)), respectively. Assumptions on contributions of AAs and insulin clearance are the same as in Model 1. Since in steady-state conditions all derivatives are equal to zero, BR_I_ is the same computed for Model 1 and described in eq. (2). The model equation is:


$$\frac{dI(t)}{dt}=k_{GL1}\cdot G(t)+k_{GL2}\cdot \frac{dG(t)}{dt}+k_{AA}\cdot AA(t)-k_{I}\cdot I(t)+BR_{I}$$

(3) $$I\text{(0)}=I_{b}$$

#### 2.1.3 Model 3

Model 3 is based on the same hypotheses of Model 2, but exponentiation (G(t)^kGL2^, with k_GL2_ dimensionless) was used to account for the contribution provided to plasma insulin by glucose fast onset, as similarly done in other studies (10):


$$\frac{dI(t)}{dt}=k_{GL1}\cdot G{\left(t\right)}^{k_{GL2}}+k_{AA}\cdot AA(t)-k_{I}\cdot I(t)+BR_{I}$$

(4) $$I(0)=I_{b}$$

BR_I_ was equal to:


(5) $$BR_{I}=-k_{GL1}\cdot G_{b}^{k_{GL2}}-k_{AA}\cdot AA_{b}+k_{I}\cdot I_{b}$$

#### 2.1.4 Model 4

Model 4 is based on the same hypothesis of Model 1, but it assumes that the transfer coefficient k_AA_ (μU·ml^-1^/(μmol·l^-1^·min)) may vary during the MMTT, thus providing a time-varying contribution:


$$\frac{dI(t)}{dt}=k_{GL}\cdot G(t)+k_{AA}(t)\cdot AA(t)-k_{I}\cdot I(t)+BR_{I}$$

(6) $$I\text{(0)}=I_{b}$$

BR_I_ was equal to:


(7) $$BR_{I}=-k_{GL}\cdot G_{b}-k_{AAb}\cdot AA_{b}+k_{I}\cdot I_{b}$$

#### 2.1.5 Model 5

Model 5 assumes that glucose contributes to plasma insulin through a compartment remote from plasma, with G_rem_(t) representing suprabasal concentration in the remote compartment; this assumption was borrowed from another model describing the kinetics of non-esterified fatty acids (11):


$$\frac{dI(t)}{dt}=k_{GL}\cdot G_{rem}(t)+k_{AA}\cdot AA(t)-k_{I}\cdot I(t)+BR_{I}$$

(8) $$I\text{(0)}=I_{b}$$

$$\frac{dG_{rem}}{dt}=-k_{GL}\cdot G_{rem}(t)+k_{GLrem}\cdot (G(t)-G_{b})$$

(9) $$G_{rem}(0)=0$$

where k_GLrem_ (min^-1^) is the glucose elimination rate from the remote compartment.

BR_I_ was equal to:


(10) $$BR_{I}=-k_{AA}\cdot AA_{b}+k_{I}\cdot I_{b}$$

#### 2.1.6 Analysis of *a priori* identifiability

*A priori* identifiability of the five competing models was tested by using DAISY (Differential Algebra for Identifiability of Systems), a software tool for structural identifiability analysis of linear and nonlinear dynamic models described by polynomial or rational ordinary differential equations with either known or unknown initial conditions (12).

### 2.2 Model implementation and parameter estimation

All models were implemented in MATLAB^®^ R2019b as ordinary differential equations (ODEs) or system of ODEs (in case of Model 5). Model solution was obtained using *ode15s*, a method for the solution of stiff differential equations. Model-parameter vector was p = [k_GL_ k_AA_], p = [k_GL1_ k_GL2_ k_AA_], p = [k_GL1_ k_GL2_ k_AA_], p = [k_GL_ k_AA_(t)], p = [k_GL_ k_GLrem_ k_AA_] for Model 1, 2, 3, 4 and 5, respectively. For each model, parameters were estimated by solving a nonlinear least-squares curve fitting problem using the *lsqnonlin* solver. Fitted data were those of insulin concentrations as described in the following section. The trust-region-reflective algorithm was specifically used, with the following lower and upper bounds set for the parameters: (0; +∞) for k_GL_, k_GL1_, k_GL2_ and k_GLrem_, and (-∞; +∞) for k_AA_ and k_AA_(t). Function and step-size tolerances were set to 10^-20^ and 10^-12^, respectively. For all models, the percent Coefficient of Variation (CV%) of the parameters estimate was computed (standard deviation (SD) of parameters estimate divided by parameters value, multiplied by 100). In addition, for the parameters with (-∞; +∞) bounds (i.e., practically unbounded), the 95% Confidence Interval (CI) was also computed. To assure convergence to a global minimum, a set of initial values for the model-parameter vector was generated and related local solutions were computed; ten different initial values for each parameter were considered (leading to 10*^npar^* attempts, where *npar* is the number of model parameters).

### 2.3 Model validation

**Figure 3:**
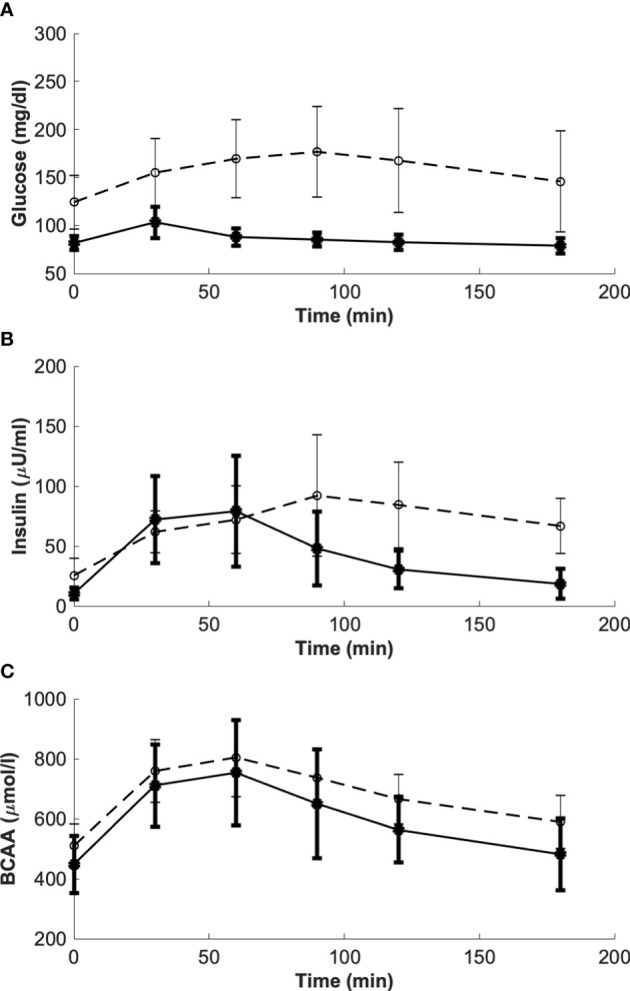
Glucose, insulin and BCAA curves from the reference mean experimental data by Karusheva et al. (7) (panels **A–C**, respectively), for glucose-tolerant participants (CNT, n=10, solid line) and patients affected by type 2 diabetes (T2D, n=10, dashed line). Closed and open circles (for CNT and T2D, respectively) indicate the time instants when the experimental samples were collected. Data are reported as mean ± SD.

The five models were initially validated using average data previously reported by Karusheva et al. (7). These data refer to glucose-tolerant (CNT, n=10) and type 2 diabetes (T2D, n=10) participants, the latter having known disease duration of less than 1 year. All participants underwent a MMTT at high protein content, thus adequate for studying AAs levels and kinetics, consisting of 378 g of the standardized commercial liquid meal Boost High Protein (Nestlé S.A., Vevey, Switzerland) for a total of 365.8 kcal (of which 9.1 g fat, 50.1 g carbohydrates, and 22.8 g protein). During the MMTT, blood sampling was performed at −10, −1, 10, 20, 30, 60, 90, 120, and 180 minutes for measurements of glucose, insulin and BCAA concentrations (7). Related temporal patterns during the MMTT are reported in **Figure 3**.

After applying all five models to the indicated average data, we selected the model that showed: (i) global *a priori* identifiability, (ii) best value in terms of corrected Akaike’s information criterion (AICc) (13), and (iii) estimated parameter plausibility. The considered AIC value was the mean of the two AIC values obtained over the CNT and T2D average data (7). For comparison purposes, the relative likelihood of each model was also considered (14). Thereafter, a second validation step was performed for the selected model considering two virtual populations, comprising 100 virtual subjects with normal glucose tolerance and 100 with T2D. Each virtual population was generated starting from the average data and SD reported by Karusheva et al. (7). The generation of the two virtual populations was accomplished by exploiting an approach already used in previous studies (15). According to this approach, in response to the MMTT each virtual subject was characterized by a curve for glucose, insulin and BCAA, respectively. Each sample of the glucose, insulin and BCAA curves was randomly generated from a normal distribution (considering all samples within the 95% CI) with mean and SD equal to those reported by Karusheva et al. (7) for the related average curve. Furthermore, in order to obtain curves that mimic the same trend of the original ones, constraints have been added during the random generation of the samples (in particular, sign of the variation between two time samples equal to that of the related average curve (7)).

### 2.4 Statistical analysis

Person’s correlation analysis was performed to analyze the relationship between k_GL_ and k_AA_ in CNT and T2D virtual populations. Regression analysis was also performed between k_GL_ and k_AA_ in the two groups pooled, with the virtual subjects’ type (CNT or T2D) as factor. By unpaired t-test, we also tested difference between CNT and T2D in both k_GL_ and k_AA_. Chi-square test was used to test differences between CNT and T2D in k_AA_ percentages of positive, negative and negligible values.

In case of skewed distributions, parameter values were log_e_-transformed before testing. Data are reported as mean ± SD unless otherwise specified. Two-sided p values less than 0.05 were considered statistically significant.

## 3 Results

The top model resulted Model 1, which was the best compromise between model parsimony and performance. Indeed, Model 1 showed global identifiability and the better AICc (20.53), as compared to other models with similar global identifiability (25.36, 25.86 and 27.07 for Model 5, 2, 3, respectively. Model 4 showed the best AICc, with very low value (-135.51) due to extremely accurate model fit, but it showed only local rather than global identifiability. Considering the relative likelihood, Model 4 would be again the best one, but for the previously mentioned identifiability issues Model 1 was preferable. When computing the relative likelihood of Model 1 *vs*. Model 2, 3 and 5, we found values remarkably higher than 1 (relative likelihood equal to 11.19, 14.39, 26.35, *vs*. Model 5, 2 and 3, respectively), this providing further support in considering Model 1 as the best model. For these reasons, we assumed that the model to be selected and hence analyzed further over the virtual populations was in fact Model 1. In any case, some results from the other models are reported in the Appendix A.

**Figure 4:**
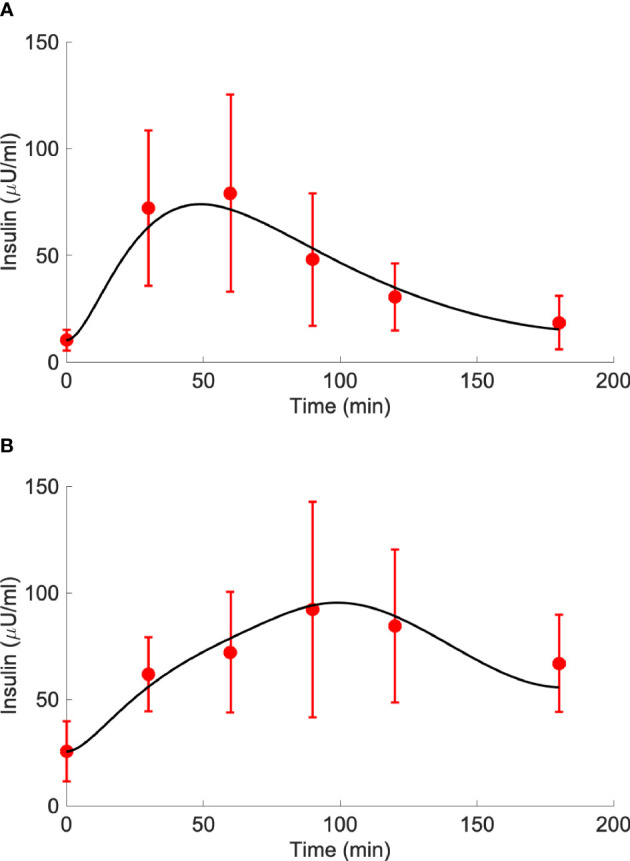
Best-fit results for model validation on reference mean experimental data by Karusheva et al. (7) for glucose-tolerant participants (CNT, n=10, panel **A**) and patients affected by type 2 diabetes (T2D, n=10, panel **B**). Red circles are the reference experimental values (mean ± SD); black lines are the model predictions.

Results of model validation on the experimental average data of the study (7) provided the best fit reported in **Figure 4**. In terms of the model parameters, for k_GL_ the estimated value was 0.0974 in CNT and 0.2087 in T2D (units: μU·ml^-1^/(mg·dl^-1^·min)); for k_AA_, the estimated value was 0.0247 in CNT and -0.0048 in T2D (units: μU·ml^-1^/(μmol·l^-1^·min)). It is worth noting that, differently from k_GL_, which has been constrained to be positive, k_AA_ was free to get possible negative values, since we could not *a priori* exclude a negligible or even deleterious effect of BCAAs on insulin secretion. Interestingly, in CNT the k_AA_ value was estimated positive (95% CI: 0.0168 – 0.0325), whereas based on the 95% CI, in T2D we obtained k_AA_ not significantly different from zero (95% CI: -0.0281 – 0.0185), suggesting a negligible contribution to insulin secretion from BCAAs.

**Figure 5:**
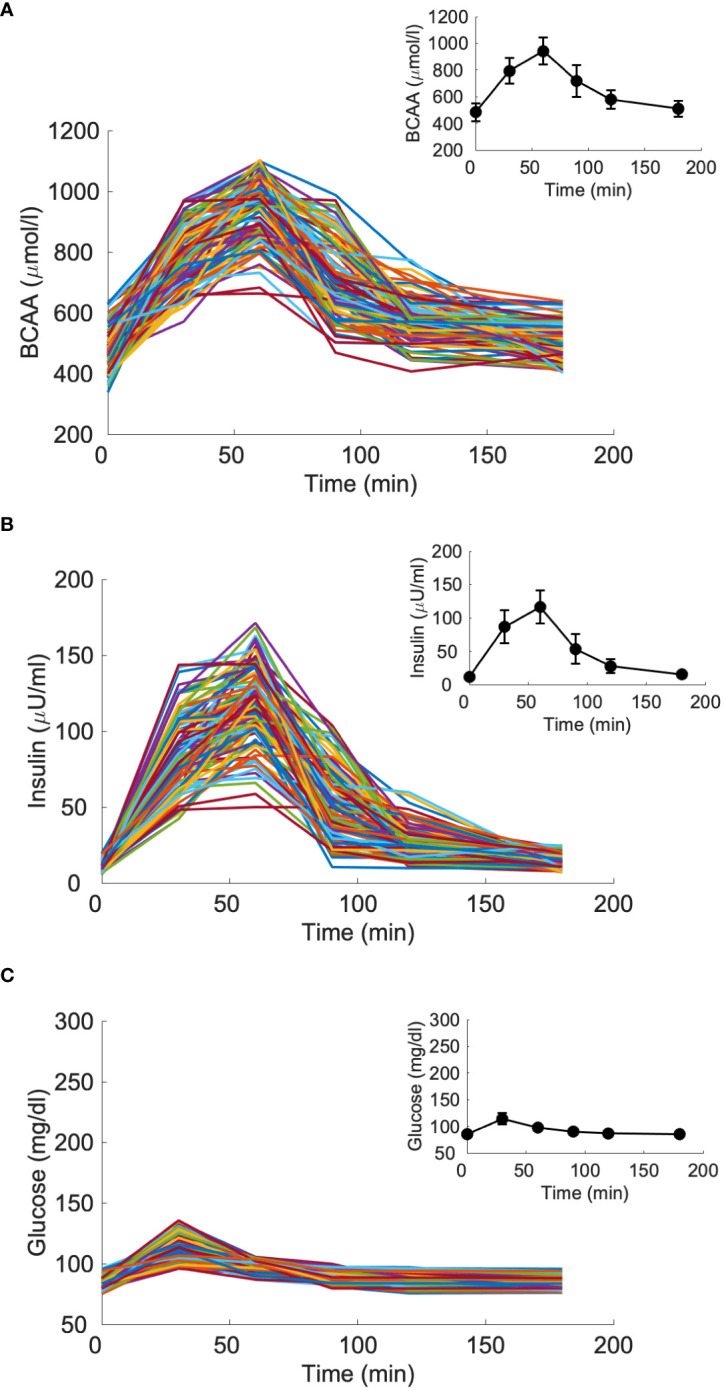
Branched-chain amino acids (BCAA), insulin and glucose curves for the virtual population composed by 100 glucose-tolerant individuals (CNT, “spaghetti plot” in panels **A**, **B**, **C**); BCAA, insulin and glucose mean (± SD) computed over the 100 curves (black lines in the inset plots).

**Figure 6:**
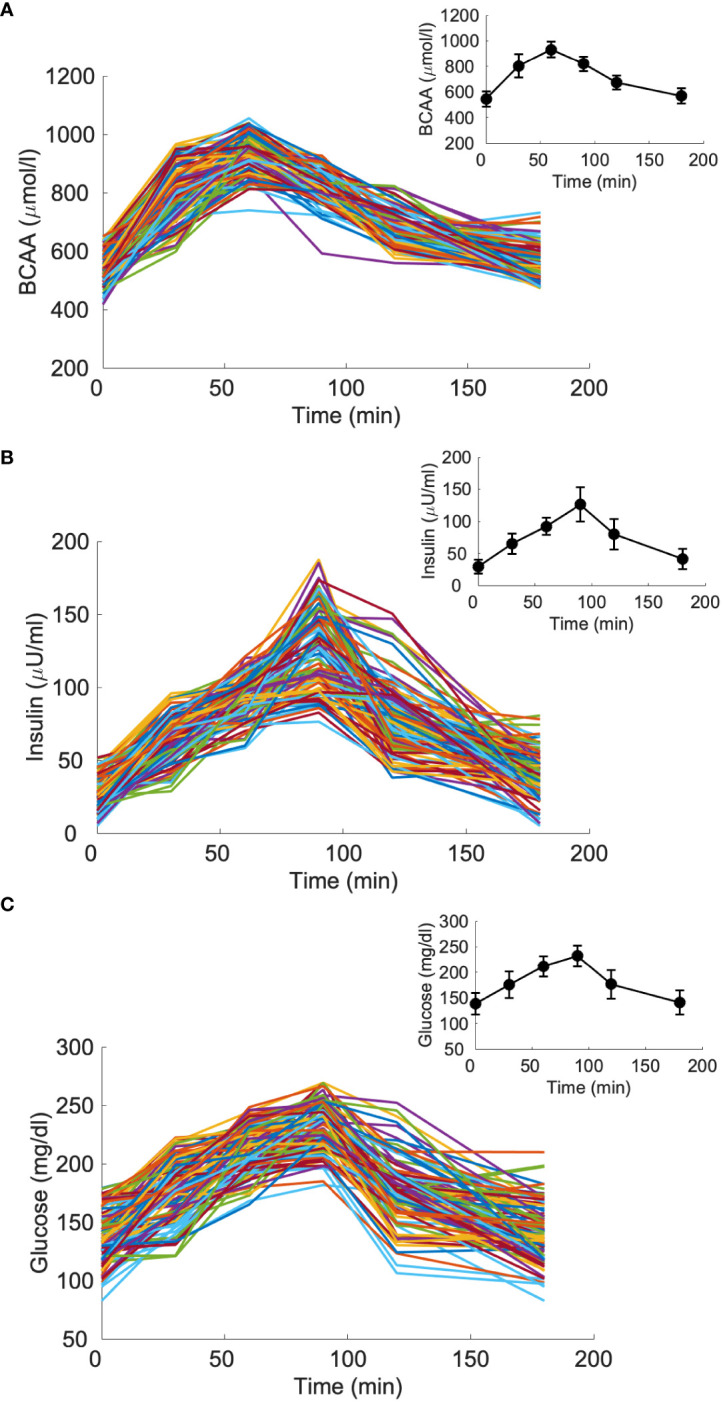
Branched-chain amino acids (BCAA), insulin and glucose curves for the virtual population composed by 100 individuals with type 2 diabetes (T2D, “spaghetti plot” in panels **A–C**); BCAA, insulin and glucose mean (± SD) computed over the 100 curves (black lines in the inset plots).

**Figure 7:**
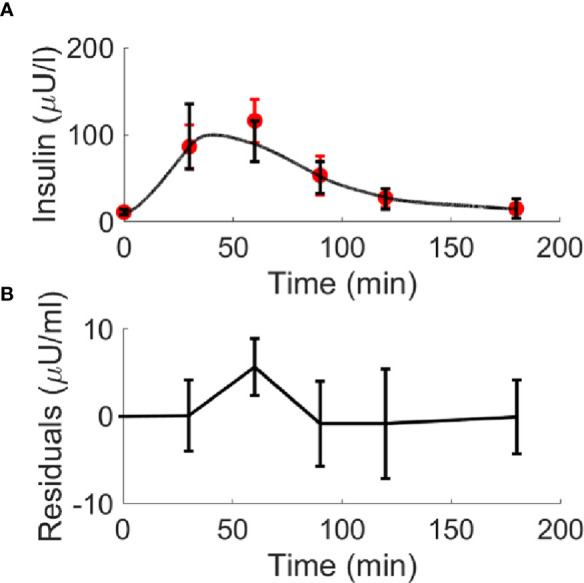
Best-fit results for model validation on the 100 virtual CNT individuals. Red circles represent the mean (± SD) over the 100 individuals, whereas black continuous line represents the mean predicted insulin curve (panel **A**); mean residuals over the 100 curves are also reported (panel **B**).

**Figure 8:**
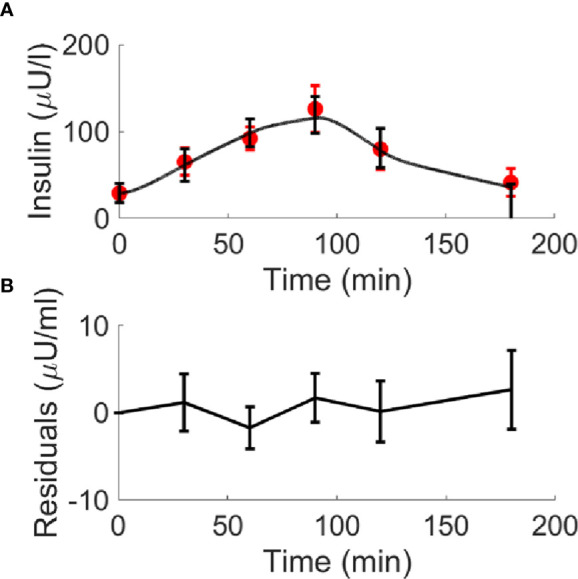
Best-fit results for model validation on the 100 virtual T2D individuals. Red circles represent the mean (± SD) over the 100 individuals, whereas black continuous line represents the mean predicted insulin curve (panel **A**); mean residuals over the 100 curves are also reported (panel **B**).

**Figure 9:**
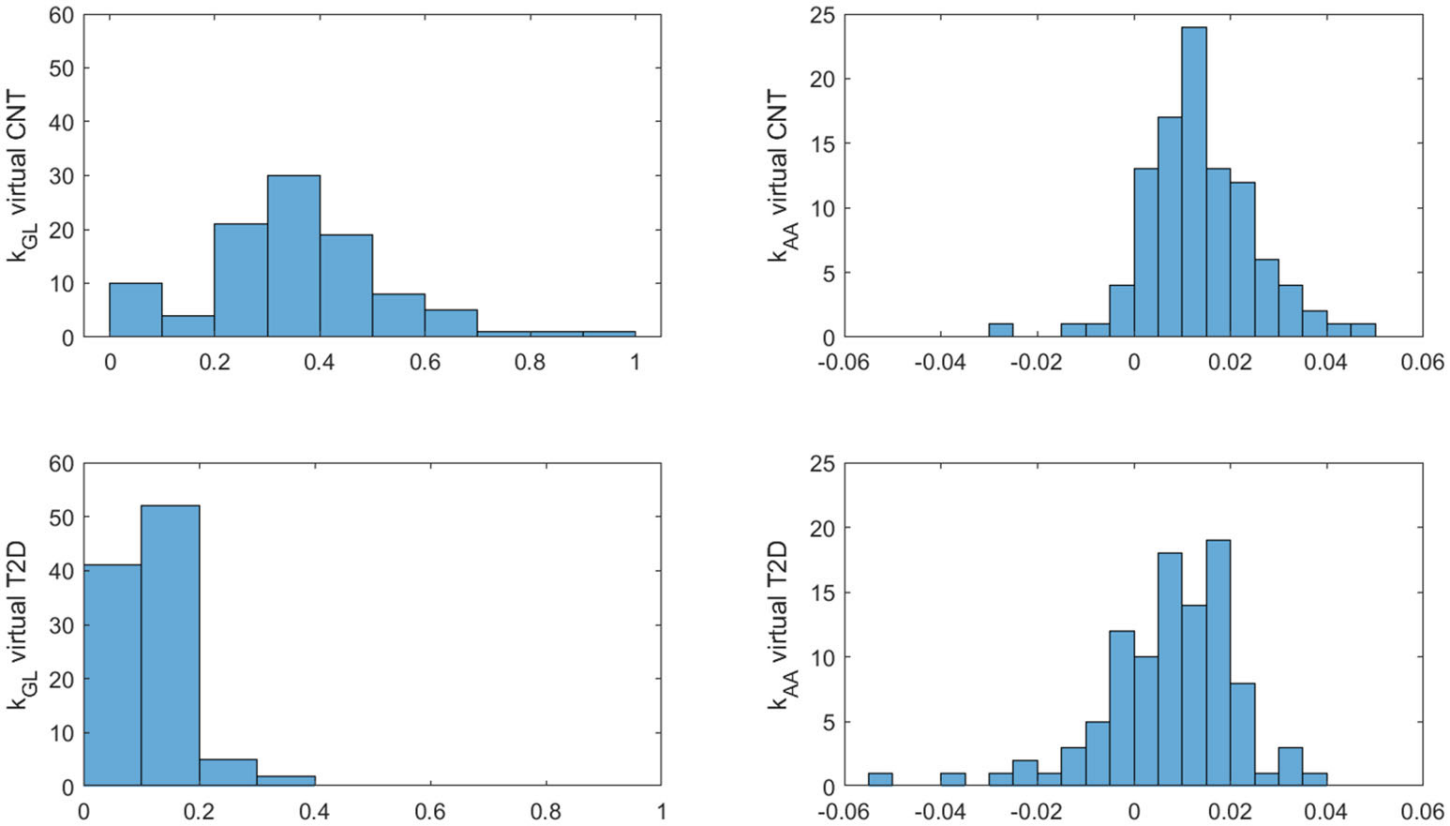
Distributions of k_GL_ and k_AA_ over the 100 virtual CNT and T2D individuals.

Insulin, glucose and BCAA curves for the two virtual populations are shown in **Figures 5**, **6**. Model validation on the two virtual populations provided the mean best fit and related residuals shown in **Figures 7**, **8**. Distributions of k_GL_ and k_AA_ over the virtual CNT and T2D subjects are reported in **Figure 9**. In terms of mean and SD, the values for k_GL_ were 0.3480 ± 0.1778 and 0.1134 ± 0.0654 μU·ml^-1^/(mg·dl^-1^·min), in CNT and T2D, respectively. In CNT, k_AA_ was 0.0134 ± 0.0112 μU·ml^-1^/(μmol·l^-1^·min), and according to the individual 95% CIs it was positive in the 53% of cases, negative in the 2%, and negligible in the 45% (i.e., CI crossing the zero value). In T2D, k_AA_ was 0.0075 ± 0.0142 μU·ml^-1^/(μmol·l^-1^·min), and it was positive in only 25% of cases, negative in the 65%, and negligible in the 10%. An inverse correlation was detected between k_GL_ and k_AA_ in CNT (r = -0.63, p<0.001) and T2D (r = -0.92, p<0.001). The inverse relationship between k_GL_ and k_AA_ was confirmed in CNT and T2D pooled (p<0.0001), with subjects’ type (CNT and T2D) being significant factor in the relationship (p<0.0001).

Furthermore, the unpaired t-test showed for both k_GL_ and k_AA_ a significant difference between virtual CNT and T2D (p<0.0001 for both parameters). In addition, we found that the percentages of positive, negative and negligible k_AA_ values (see percentage values reported above) were different between the two groups (p=0.0015 or lower). Consistently, in CNT the percentage of positive k_AA_ values was higher than that of negative values, whereas in T2D it held the opposite condition (p<0.0001 for both tests).

## 4 Discussion

In this study, we propose a modelling approach to determine effects of AAs on insulin secretion in humans with normal glucose tolerance and T2D. The importance of our model strategy lies in the opportunity to estimate in single individuals the possible effects of AAs on insulin secretion, in addition to the known role of glucose. Specifically, the model assesses AAs effects on insulin secretion under dynamic (stimulated) conditions, but on the other hand physiologically consistent, as determined by food ingestion. The model includes glucose as the major determinant of insulin secretion, but also AAs during the mixed-meal ingestion. The k_AA_ model parameter in the single person may then be seen as the individual sensitivity of the beta cell to AAs stimulation (in short, beta-cell AAs sensitivity).

Based on the experimental data in the glucose tolerant people that we exploited for our model development (7), we found a significant contribution of AAs in explaining insulin secretion (i.e., positive k_AA_, with positive 95% CI). Instead, in T2D, the model essentially indicated a negligible k_AA_ (CI including zero), and hence negligible contribution of AAs to insulin secretion. This result is consistent with that of the study by Karusheva et al. (7) in T2D (based on the analysis of the patients’ experimental data without model support). With regard to the virtual population, we found that in the control participants k_AA_ was positive in more than half of cases, whereas in T2D k_AA_ was positive only in 1/4 of cases. Accordingly, we found that k_AA_ was higher in virtual CNT than T2D. We also found that the relationship between k_AA_ and k_GL_ was significant, but different between the two virtual groups. These results need further confirmation in future studies on real data, but they appear reasonable and physiologically plausible, thus further suggesting appropriateness and reliability of our modelling approach.

One aspect of our results that, in contrast, appears somehow questionable relates to the k_GL_ values in the experimental data. Indeed, k_GL_ was lower in the CNT than in T2D. However, it has to be considered that the average data in each of the two participants’ group represent kind of further single subject, different from the real ones. On the other hand, the virtual populations analysis showed that parameters value distributions in the two groups partially overlap, thus meaning that there are in fact some virtual CNT subjects with k_GL_ lower than in some virtual T2D subjects. This aspect emphasizes the relevance of the virtual analysis as well. Thus, the indicated result, found in the experimental-based average data analysis, is not unaccountable. It is also worth noting that the lower k_GL_ in CNT than in T2D appears compensated by the higher k_AA_, which is significant in CNT while negligible in T2D.

The remarkable importance of studying the stimulatory effect of AAs on insulin secretion has been recognized already decades ago (1, 16–18). Among the AAs, arginine has been clearly shown as a potent insulin secretagogue (18, 19). Notably, the insulinotropic effect of arginine was shown in both normal glucose tolerance (20) and T2D (21). On the other hand, apart for arginine, the possible insulinotropic effect of specific AAs has been investigated in several studies. Already in one of the earlier studies, it was reported that in healthy people intravenous administration of leucine was able to induce a robust release of insulin that conversely was not observed when injecting a solution of eight amino acids not containing leucine (22). Those findings were confirmed and corroborated some years later in an animal model by another study, which analyzed the effects of intravenous infusion of 17 amino acids. It was found that leucine was the most effective amino acid in stimulating insulin secretion, followed by alanine, glycine, and serine (23). Generally, it was shown that neutral straight-chain amino acids stimulated both insulin and glucagon secretion, with greater secretory response than shorter C-chain amino acids, whereas branched-chain amino acids tended to enhance insulin and suppress glucagon secretion (23). Another study showed in a perfused rat pancreas model that, in the presence of glucose, first-phase insulin secretion was potentiated by both cationic AAs (arginine and lysine) and BCAAs (leucine). Conversely, arginine and lysine determined inhibition of second-phase insulin secretion, whereas leucine had no significant inhibitory effect, this suggesting that those amino acids mediate their effects on first- and second-phase insulin release *via* different mechanisms of action, which may reflect differences in charge and/or metabolic fates within the beta cell (24). The strong insulinotropic characteristics of leucine were also reported in other studies, as outlined in a review report (25). Some studies also analyzed the increase in insulin response following AAs ingestion (with special focus on leucine) in both healthy control and T2D subjects (26, 27). The increase in insulin response was observed in both control and T2D groups, but, interestingly, in T2D such increase was typically more robust, with insulin release nearly tripled following the AAs ingestion (26).

The indicated review (25) also emphasized that, beyond the insulinotropic action, leucine may increase AAs availability for muscle protein synthesis and reduce muscle protein breakdown, this being important for prevention of muscle-related syndromes, such as sarcopenia (25). This may be of special relevance in T2D, in relation to the bidirectional relationship between T2D and sarcopenia (28, 29), remarkably linked to nutritional aspects including AAs supplementation (30, 31). It should also be observed that AAs may potentiate insulin secretion through indirect mechanisms involving incretin hormones. In fact, the effect of incretins in promoting insulin secretion has been clearly established (32, 33), and accurately quantified by appropriate methodology (34, 35). Interestingly, one study investigated the effects of combined AAs and incretin hormones administration by performing experiments in healthy humans with intravenous infusion of an AA mixture, and of human GIP or GLP-1-(7–36) amide, and found that AAs affected insulin secretion indirectly, by improving the potentiation of insulin secretion determined by GIP and GLP-1 (36).

The wide set of studies on the possible effect of AAs on insulin secretion and kinetics emphasizes the heterogeneity of the AAs action on insulin, which may involve several different mechanisms (direct and indirect), may be different for the different AAs, and may depend on the phenotypic characteristics of the studied subjects (for instance, their glucose tolerance category). This may explain the different findings of some studies, which at first sight may appear contradictory. In fact, one study in Chinese healthy subjects showed that, following a MMTT, fasting and postprandial plasma BCAA levels were consistently associated with greater fasting and postprandial insulin secretion (5). Instead, in the study from our research group, similarly based on a MMTT (7), in T2D we found postprandial isoleucine higher than in controls, but neither isoleucine nor the other BCAAs correlated with C-peptide levels (assumed as marker of insulin secretion). On the other hand, it should be noted that in another study in T2D, we found that short-term dietary reduction of BCAAs decreased postprandial insulin secretion (6). In addition, in the study (7), we also investigated a group of type 1 diabetic patients, and found increased BCAA levels that were associated with lower C-peptide (i.e., lower insulin secretion). Nonetheless, as stated above, we hypothesize no contradiction between those studies (5–7), as the observed differences in the association between BCAAs and insulin secretion among those studies are likely mainly due to the different categories of the subjects under investigation (healthy, type 1 and type 2 diabetes), and partly to the different experimental conditions and study purposes. Since our proposed model may be applied to data related to different AAs and different categories of patients (ranging from normal glucose tolerance to type 2 diabetes), we are confident that it will contribute to clarify the possible specific effects of various AAs on insulin secretion, also likely depending on the studied population. Notably, single AA may be also analyzed, in the hypothesis that the study providing the AA data has appropriate design. As an example, the model can be applied to the analysis of data derived from experiments consisting in administration of a glucose-leucine mixture (37). In Appendix B we reported some results related to the analysis of the glucose-leucine data of that study (37).

Several mathematical models describing the effects of different compounds on insulin secretion and beta-cell function have been proposed (38). Surprisingly, in contrast to other compounds carefully studied with mathematical models (especially the incretin hormones), for AAs we found only one model pertinent to our scientific literature analysis (8), despite the wide cluster of experimental studies showing effects of AAs on insulin secretion, as outlined above. This further motivated us to develop the models presented in this study. Of note, in the indicated previous model (8), the main aim was to gain insights into the role played by alanine in both amino acid-stimulated insulin secretion and in glucose stimulated insulin secretion. The model described the kinetics of core metabolic processes leading to ATP production and Ca^2+^ handling in the pancreatic β-cells, and related these processes to insulin secretion. Experimental data were collected over a rat insulin-secreting cell line and used for validating the model and for fine-tuning of the parameters. The model simulations suggested that alanine produces a potent insulinotropic effect *via* both a stimulatory impact on beta-cell metabolism, and as a result of the membrane depolarization due to Ca^2+^ influx triggered by alanine and Na^+^ co-transport. In summary, the model (8) was developed for simulation purposes aimed at the investigation of the mechanisms responsible for the AAs effects on the beta cell, in an *in-vitro* context. Our model aim, instead, is the assessment of the possible effects of AAs in single individuals, in an *in-vivo* context, with no expectation to get insight into cellular mechanisms. Thus, given the major differences in terms of approach and purposes, no real comparison is possible between the model (8) and our model.

In this study, we analyzed the performance of different models, and eventually selected the specific model showing the best balance between accuracy in model fit, parsimony in the number of model parameters, and plausibility of the model parameter values. Of note, in some cases only the equations describing glucose behavior were changing among models, but nonetheless we were expecting effects on both glucose and AAs parameters. Indeed, each model somehow “distributes” to glucose and AAs part of the effect on the output variable (insulin). Thus, changing the formulation of the glucose model section would affect the assessment of both glucose and AAs effect on insulin, and vice-versa.

We finally selected the model that showed the best corrected Akaike’s information criterion (AICc) value (13) except for the model with time variant k_AA_ parameter. In fact, the time variant model yielded the best AICc (very low value), but our analysis of model identifiability indicated the model as locally and not globally (i.e., univocally) identifiable. This means that the model parameters can assume a limited set of values, which may still be acceptable under certain circumstances, but it represents a potential weakness of the model. Thus, since Model 1 showed the best AICc compared to all remaining models, it was globally identifiable, and provided reasonable estimated parameter values, we selected it as the most appropriate and convenient for our applications. Nonetheless, we reported in Appendix A the formulation of the excluded models, since they may be useful for possible future studies in other populations or in different experimental conditions. Notably, the time-variant model may be improved by possible application of regularization strategies, as done in some of our previous studies (15, 34). It is also worth noting that we tested both a two-step and a single-step modelling approach. In the two-steps approach, we first run the model without AAs contribution, thus determining the effect of glucose on insulin secretion. In the second step, AAs was introduced, thus allowing AAs to yield the residual contribution for the full explanation of insulin secretion, if needed. Instead, in the one-step approach both glucose and AAs role are estimated concomitantly. In terms of modelling strategy both approaches have potential advantages and pitfalls, but we eventually selected the one-step approach being more consistent with the considered physiological processes, because both glucose and AAs are expected to act concomitantly on insulin release and kinetics.

Our study has some limitations. First, the model is currently not applicable in people with type 1 diabetes, because it has not been yet validated using type 1 diabetes data. Indeed, the different findings of the study (7) with regard to type 2 and type 1 diabetes indicated that the two types of diabetes may require somehow customized modelling approach. Thus, we agreed that for the moment it was convenient to focus only on T2D. As a matter of fact, the model should not be used in data from populations with metabolic abnormalities different from simple impaired glucose regulation (prediabetes) or overt T2D. Another limitation may be seen in the fact that the experimental data used for modelling validation was restricted to average rather than individual AAs data. However, this was counteracted by the extensive analysis in virtual subjects (generated with wide variations around the experimental reference AA values of study (7)), this amending for the possible limitation. Of note, we have been permissive in the generation of the virtual patients (to avoid possible unphysiological constraints), but on the other hand this provided some cases with particularly high sample values (i.e., hardly observable in real subjects). This may explain the imperfect fit in those cases, as also observable in the average fit at the high insulin values in the virtual subjects with normal glucose tolerance. Furthermore, another limitation of the study is that the selected model, as conceived, determines the AAs effect on insulin kinetics rather than on insulin secretion and insulin clearance separately. On the other hand, the scientific literature on the AAs contribution to insulin kinetics rarely showed AAs effects on insulin clearance, and hence we are confident that the insulin kinetics effects described by our model are mainly attributable to insulin secretion, rather than to insulin clearance.

Furthermore, in the present model we did not consider the potentiating effect of incretin hormones on insulin secretion, which was instead analyzed in several previous models from both our research group and others, as recently reviewed (38). This is a limitation, but it was necessary to avoid excessive model complexity, which would prevent applicability in the clinical context. In fact, currently it is not extremely common that metabolic datasets include glucose, insulin and amino acids measures, though we expect that more datasets will be available in the future for clarification of still unsolved aspects of individual amino acids actions, in the light of the general recommendations for precision medicine in metabolism and diabetes (39). Thus, metabolic datasets including incretin hormones measure in addition to that of glucose, insulin and amino acids are even rarer. Developing a model requiring incretin hormones in addition to glucose, insulin and amino acids would render a model hardly usable in the clinical practice. Since potential clinical applicability of our model was a pivotal goal of the study, we did not explicitly model the incretin effect. It has also to be noted that the model is not overfitted (model residual has shown to be not high but not negligible either). Thus, we expected that the effect of incretin hormones, and possible other variables not explicitly modelled that may affect insulin secretion, is likely captured (i.e., included) in the model residuals. On the other hand, we acknowledge that we cannot exclude a bias in the determination of the model parameters, though we hypothesize it modest for the reasons explained. This is especially true in the case of experiments with robust administration of proteins (including amino acids), as in the MMTT originating the data that we have first analyzed (7).

It should also be acknowledged that such MMTT (Boost High Protein, Nestlé S.A., Vevey, Switzerland) included several amino acids (i.e., not only BCAAs). Since we analyzed BCAAs only, the possible effect of the other AAs was not taken into account. However, similarly to the incretin effect, as reported above we expect the possible effect of other AAs being likely captured in model residuals.

Finally, some caveats are necessary to prevent misuse of the model. In fact, when exploiting our model to analyze the possible effect of one AA (or a pool of AAs, as in this study), it is necessary to ensure that the study design is adequate for our purposes. The studied AA/AAs must be appropriately stimulated by the experimental procedure, thus determining sufficient variation from the basal, unstimulated value. In addition, it should be already established from previous knowledge that the AA/AAs under investigation can affect insulin secretion. Furthermore, the experiments should ensure that the effect of the AA/AAs of interest is not much lower than that of other AAs or different compounds. Indeed, in such conditions the model would hardly be able to reliable “extract” from the data the specific small effect of the AA/AAs under analysis.

In conclusion, this study proposes a model to determine the effects of amino acids on insulin secretion in people with or without type 2 diabetes. The model provides the opportunity to estimate amino acids-induced insulin secretion in single individuals, possibly contributing to setting up patient’s tailored dietary prescriptions to delay the onset of diabetes or prevent further metabolic derangement in patients already suffering from diabetes.

## Data availability statement

The original contributions presented in the study are included in the article/**Supplementary Material**. Further inquiries can be directed to the corresponding author.

## Author contributions

MM, MCP, and AT conceived and designed the study. MM, MCP, and AT analyzed and interpreted the data. CG, LB, YK, MR, and GP validated the analysis. MM and AT wrote the first draft of the manuscript. MCP, CG, LB, YK, MR, and GP critically revised the manuscript. All authors contributed to the article and approved the submitted version.

## Funding

This study was partly funded by a research contract for a scientific project commissioned to the CNR Institute of Neuroscience (Padova, Italy) by the German Diabetes Center (Düsseldorf, Germany) (April 2018 – April 2019; CNR protocol no. 0001219). The experimental average data by YK et al. (7) that we analyzed in the present study derives from the German Diabetes Study, initiated and financed by the German Diabetes Center, which is funded by the German Federal Ministry of Health (Berlin, Germany), the Ministry of Culture and Science of the state North Rhine-Westphalia (Düsseldorf, Germany), and the German Federal Ministry of Education and Research (Berlin, Germany). The study by YK et al. (7) was also supported by grants of German Research Foundation (DFG, CRC 1116/2) and of the Schmutzler Stiftung.

## Conflict of interest

The authors declare that the research was conducted in the absence of any commercial or financial relationships that could be construed as a potential conflict of interest.

## Publisher’s note

All claims expressed in this article are solely those of the authors and do not necessarily represent those of their affiliated organizations, or those of the publisher, the editors and the reviewers. Any product that may be evaluated in this article, or claim that may be made by its manufacturer, is not guaranteed or endorsed by the publisher.
