# Supplementary material for: Mathematical model of insulin kinetics accounting for the amino acids effect during a mixed meal tolerance test
Source: Front Endocrinol (Lausanne). 2022 Sep 15;13:966305. doi: 10.3389/fendo.2022.966305 (PMC9519856; doi:10.3389/fendo.2022.966305)
Supplement: Supplementary file 1 [file Presentation_1.pdf]

Supplementary Material

1 Appendix A

In this appendix, some results concerning the other competing models, compared to Model 1, are provided. The a-priori identifiability analysis resulted in global identifiability for Model 2, 3 and 5 and in local identifiability for Model 4. Results of model validation on the experimental average data of the study by Karusheva et al. (7) provided the best fit reported in Figures A1, A2, A3 and A4; related values of model parameter estimates are reported in Table A1. As for Model 4, $k_{AA}(t)$ remained quite stable over time, thus making Model 4 similar to Model 1. On the other hand, reliability of parameters estimate in Model 4 is not satisfactory, as shown by very high CV% (see Table A1).

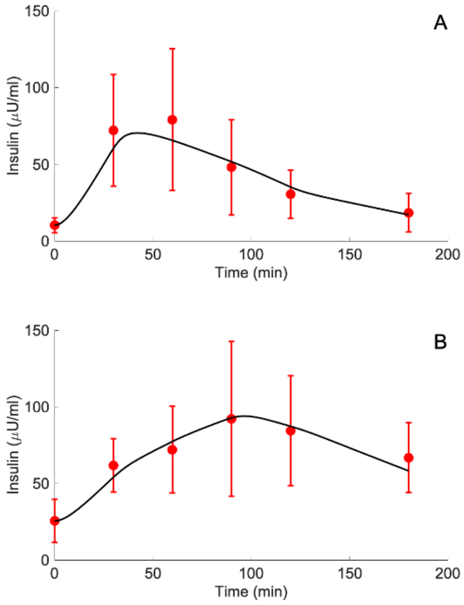

Supplementary Figure A1. Best-fit results for validation of Model 2 on reference mean experimental data by Karusheva et al. (7) for glucose-tolerant subjects (CNT, n=10, panel A) and a group of subjects affected by type 2 diabetes (T2D, n=10, panel B). Red circles are the reference experimental values (mean ± SD); black lines are the model predictions.

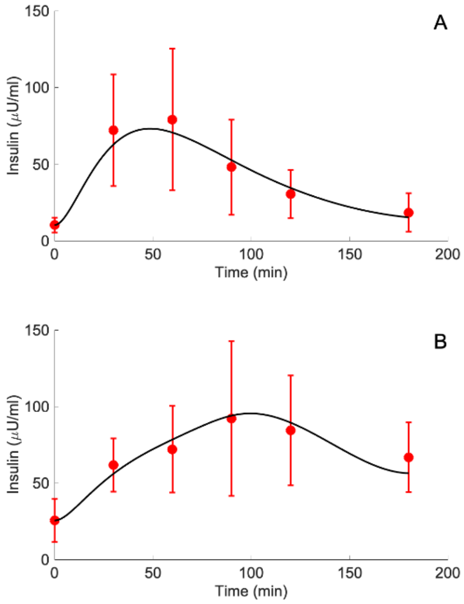

Supplementary Figure A2. Best-fit results for validation of Model 3 on reference mean experimental data by Karusheva et al. (7) for glucose-tolerant subjects (CNT, n=10, panel A) and a group of subjects affected by type 2 diabetes (T2D, n=10, panel B). Red circles are the reference experimental values (mean ± SD); black lines are the model predictions.

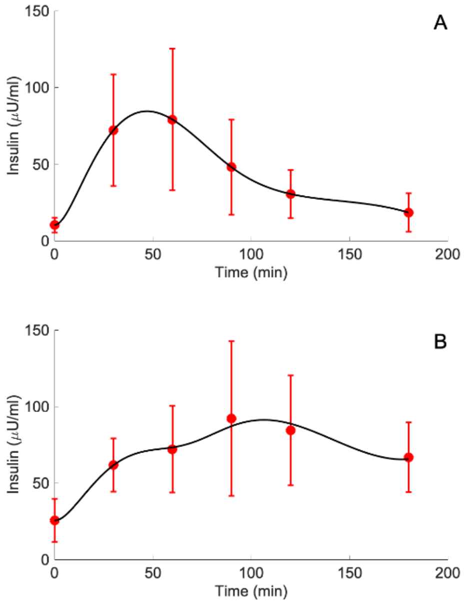

Supplementary Figure A3. Best-fit results for validation of Model 4 on reference mean experimental data by Karusheva et al. (7) for glucose-tolerant subjects (CNT, n=10, panel A) and a group of subjects affected by type 2 diabetes (T2D, n=10, panel B). Red circles are the reference experimental values (mean ± SD); black lines are the model predictions.

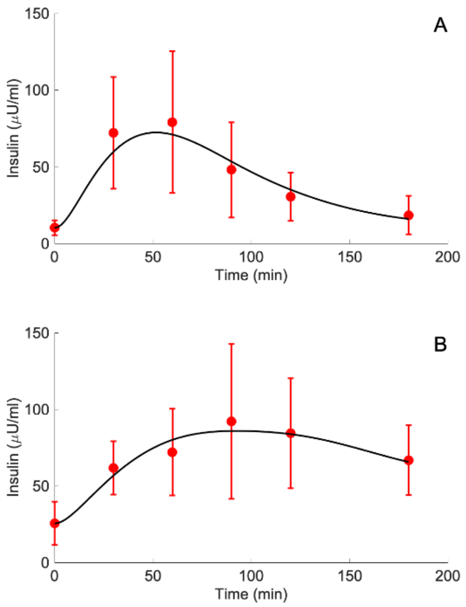

Supplementary Figure A4. Best-fit results for validation of Model 5 on reference mean experimental data by Karusheva et al. (7) for glucose-tolerant subjects (CNT, n=10, panel A) and a group of subjects affected by type 2 diabetes (T2D, n=10, panel B). Red circles are the reference experimental values (mean ± SD); black lines are the model predictions.

Table A1. Values and percent Coefficient of Variations (CV%) for parameter estimates of Model 1, 2, 3, 4 and 5 on reference average experimental data by Karusheva et al. (7). For Model 4, $k_{AA}(t)$ was estimated in the time points t equal to 0, 10, 20, 30, corresponding to k_{AA1}, k_{AA2}, k_{AA3} and k_{AA4} respectively (in the successive time points $k_{AA}(t)$ was assumed equal to k_{AA4}). The coefficient of determination (R^2) of the models fit, and related p value, are also reported.

CNT				T2D			
R^2, p	**Estimated parameter**	**Value**	**CV%**	R^2, p	**Estimated parameter**	**Value**	**CV%**
**MODEL 1**							
R^2=0.9771, p=0.00020	$\mathbf{k_{GL}}$	0.0974	17.7%	R^2=0.9157, p=0.00274	$\mathbf{k_{GL}}$	0.2087	8.3%
	$\mathbf{k_{AA}}$	0.0247	3.1%		$\mathbf{k_{AA}}$	-0.0048	62.9%
**MODEL 2**							
R^2=0.9787, p=0.00017	$\mathbf{k_{GL1}}$	0.0331	<1.0%	R^2=0.9380, p=0.00147	$\mathbf{k_{GL1}}$	0.2173	2.9%
	$\mathbf{k_{GL2}}$	2.0101	13.3%		$\mathbf{k_{GL2}}$	0.2635	8.0%
	$\mathbf{k_{AA}}$	0.0267	2.9%		$\mathbf{k_{AA}}$	-0.0069	<1.0%
**MODEL 3**							
R^2=0.9785, p=0.00017	$\mathbf{k_{GL1}}$	0.0065	<1.0%	R^2=0.9226, p=0.00274	$\mathbf{k_{GL1}}$	1.0104	7.7%
	$\mathbf{k_{GL2}}$	1.5078	2.3%		$\mathbf{k_{GL2}}$	0.7469	1.5%
	$\mathbf{k_{AA}}$	0.0245	3.2%		$\mathbf{k_{AA}}$	-0.0057	<1.0%
**MODEL 4**							
R^2=1.000, p<0.00001	$\mathbf{k_{GL}}$	0.1121	678.1%	R^2=0.9848, p<0.00001	$\mathbf{k_{GL}}$	0.1773	10.1%
	$\mathbf{k_{AA1}}$	0.0160	226.9%		$\mathbf{k_{AA1}}$	-0.0195	7.7%
	$\mathbf{k_{AA2}}$	0.0209	223.9%		$\mathbf{k_{AA2}}$	-0.0124	5.6%
	$\mathbf{k_{AA3}}$	0.0203	134.5%		$\mathbf{k_{AA3}}$	-0.0138	<1.0%
	$\mathbf{k_{AA4}}$	0.0171	173.9%		$\mathbf{k_{AA4}}$	-0.0138	5.8%
**MODEL 5**							
R^2=0.9733, p=0.00027	$\mathbf{k_{GL}}$	0.7982	34.1%	R^2=0.9592, p=0.00063	$\mathbf{k_{GL}}$	0.0210	32.9%
	$\mathbf{k_{GLrem}}$	0.0696	35.6%		$\mathbf{k_{GLrem}}$	0.1255	8.2%
	$\mathbf{k_{AA}}$	0.0252	4.0%		$\mathbf{k_{AA}}$	0.0175	11.9%

2 Appendix B

In this appendix, results of Model 1 validation on single AA administration are provided. Validation was performed by fitting Model 1 on mean data reported in the study by Kalogeropoulou et al. (37), in which a group of thirteen healthy participants underwent a meal test consisting of 1 mmol leucine per kilogram lean body mass plus 25 g glucose. Blood sampling was performed every 10 minutes for 120 minutes and then at 150 minutes, for the measure of glucose, insulin and leucine concentration. Results of model validation provided the best fit reported in Figure B1, with k_{GL} equal to 0.1261 $\mu U \cdot ml^{-1}/(mg \cdot dl^{-1} \cdot min)$ and k_{AA} equal to 0.0040 $\mu U \cdot ml^{-1}/(\mu mol \cdot l^{-1} \cdot min)$ (95% CI: 0.0032 – 0.0049).

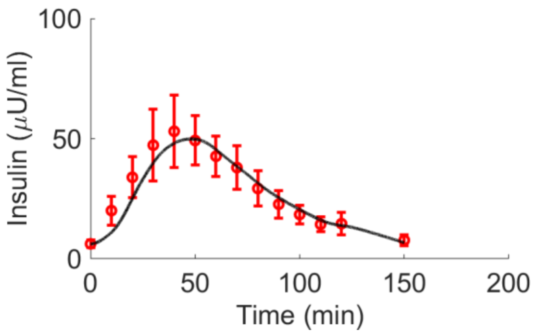

Supplementary Figure B5. Best-fit results for validation of Model 1 on mean experimental data by Kalogeropoulou et al. for healthy subjects (n=13). Red circles are the reference experimental values (mean ± standard error of the mean, SEM); black lines are the model predictions.
